# Generation shifts in smoking over 20 years in two Dutch population-based cohorts aged 20–100 years

**DOI:** 10.1186/s12889-015-1481-3

**Published:** 2015-02-13

**Authors:** Enrico Raho, Sandra H van Oostrom, Marjolein Visser, Martijn Huisman, Else M Zantinge, Henriette A Smit, WM Monique Verschuren, Gerben Hulsegge, H Susan J Picavet

**Affiliations:** Centre for Nutrition, Prevention and Health Services, National Institute for Public Health and the Environment, Bilthoven, The Netherlands; Department of Health Sciences, Faculty of Earth and Life Sciences, VU University, Amsterdam, The Netherlands; EMGO+ Institute for Health and Care Research, Department of Epidemiology & Biostatistics, VU University Medical Center, Amsterdam, The Netherlands; Department of Sociology, VU University, Amsterdam, The Netherlands; Centre for Health and Society, National Institute for Public Health and the Environment, Bilthoven, The Netherlands; Julius Center for Health Sciences and Primary Care, University Medical Center Utrecht, Utrecht, The Netherlands

**Keywords:** Generation shifts, Smoking, Education, Prospective cohort study

## Abstract

**Background:**

Younger and older generations may differ substantially in their lifetime smoking habits, which may result in generation-specific health challenges. We aimed to quantify generation shifts in smoking over a period of 25 years.

**Methods:**

We used the Doetinchem Cohort Study (baseline 1987–1991; 7768 individuals; 20–60 years; follow-up 1993–2012) and the Longitudinal Aging Study Amsterdam (baseline 1992–1993; 3017 individuals; 55–85 years; follow-up 1995–2009). Generation shifts were studied between 10-year generations (age range: 20–100 years). Generation shifts were examined graphically and by using logistic random effect models for men and women.

**Results:**

Among men, significant generation shifts in *current smoking* were found between two non-successive generations: for instance men in their 40s at baseline smoked much more than men in their 40s at follow-up (33.6% vs. 23.1%, p < 0.05). Among women, the most recently born generation showed a favourable significant generation shift in *current smoking* (−7.3%) and *ever smoking* (−10.1%). For all other generations, the prevalence of *ever smoking* among women was significantly higher in every more recently born generation, whereas no other generation shifts were observed for *current smoking*. The unfavourable generation shifts were mainly found among the lower educated.

**Conclusions:**

The future burden of disease due to smoking is expected to be reduced among men, but not yet among women. Educational differences in smoking-related health problems are expected to increase.

## Background

General smoking prevalence rates hide differences in smoking between generations, sexes and socio-economic groups [1,2]. Negative health effects due to smoking depend in particular on long-term exposure, and this might differ substantially between younger and older generations of men and women. For a few countries other than the Netherlands it was already shown that the prevalence of current smoking was lower in younger generations as compared to older generations at the same age [3-12]: especially in men younger generations smoked less often than their predecessors, while in women more unfavourable trends in smoking were observed since women born between 1950 and 1980 smoked more often than their predecessors.

Studies on smoking trends and especially changes in smoking behaviour between generations are rare, but these insights are needed for forecasts of health and disease. Besides sex-specific analyses, it is interesting to study generation shifts in different educational groups. Few studies suggest that educational inequalities regarding smoking are widening with the low educated doing worse [6,7,12,13].

Using two long-running population-based cohort studies, the objective of this study was to describe trends in smoking behaviour in different generations that were followed over time. The Doetinchem Cohort Study (DCS) and the Longitudinal Aging Study Amsterdam (LASA) together represent a wide age-range (20–100 years) of Dutch men and women with a follow-up of 25 and 17 years, respectively. Differences, over time, in the prevalence of current and ever smoking between successive and non-successive 10-year generations were described by sex and attained level of education.

## Methods

### Study populations

The Doetinchem Cohort Study (DCS) is a population-based cohort study focused on the impact of (changes in) lifestyle factors and biological risk factors on health while ageing [14]. Based on an age- and sex-stratified sample survey from the civil registries, 20155 inhabitants of the Dutch town Doetinchem were invited to participate in the first examination wave between 1987 and 1991. From the participants in this first wave (n = 12405, response rate 62%), a random sample of 7768 persons (aged 20–59 years) was re-invited every five years for in total five subsequent waves until 2012. Participants were not re-invited when they did not give permission to retrieve their information from the municipal administration, when they missed two examinations in a row, emigrated, actively withdrew from the study, or died. Response rates for the second to fifth wave were 79%, 75%, 78% and 78%, respectively. The study was approved by the external Medical Ethics Committee of the Netherlands Organization of Applied Scientific Research Institute and the University of Utrecht according to the guidelines of the Helsinki Declaration. All participants gave written informed consent. The DCS study is described in detail elsewhere [14].

The Longitudinal Aging Study Amsterdam (LASA) is a population-based cohort study, which aims to study the impact of physical, cognitive, emotional and social functioning in relation to ageing [15]. A random sample of older men and women (aged 55–84 years, N = 4494), stratified by age and gender, degree of urbanization, and expected 5-year mortality, was drawn from the population registries of 11 Dutch municipalities in the geographical areas of Amsterdam, Oss, and Zwolle, with an oversampling of older people and older men in particular. These three regions were selected to achieve an optimal representation of the older Dutch population. Data collection started in 1992–1993 (response rate 69%, n = 3107). Follow-up measurements were performed every three years for in total six subsequent waves between 1992 and 2009. Response rates for the second to sixth wave were 82%, 82%, 81%, 75% and 78%, respectively. Cumulative attrition during the 16 years of follow-up in LASA was mainly due to death and to a lesser extent caused by refusal and frailty or no establishment of contact. Informed consent was obtained from all participants and the Ethical Review Board of the VU University Medical Centre approved the study. The LASA study is described in detail elsewhere [15].

### Measures

We focused on the prevalence of current smoking and ever smoking of cigarettes. We excluded smokers of pipes or cigars only, since 95% or more of the smokers in the cohorts consisted of cigarettes smokers. Information on smoking was assessed by questionnaire in the DCS and during a medical interview in LASA. ‘Did you ever smoke regularly?’ was asked in order to define ever and never-smokers. Ever smokers were then asked if they were a current smoker or an ex-smoker (‘Do you smoke (at present)?’). Additionally, ever smokers were asked whether they smoked cigarettes.

Level of education was measured in the DCS as the highest level reached during the time of study and in the LASA as the highest level reached at the time of the baseline measurement. Level of education was classified in three categories: low education includes education up to lower vocational education, middle education ranges from general intermediate to general secondary education, and a higher educational level includes higher vocational education up to scientific/university education.

### Statistical analyses

Generations were defined based on the age at baseline of the participants. Four generations were defined in the DCS (20–29, 30–39, 40–49 and 50–59 years) and four generations in LASA (55–59, 60–69, 70–79 and 80–89 years). Successive generations had a chronological age range, for example 30–39 and 40–49. Non-successive generations had an age range with 10 years in between, for example 30–39 and 50–59.

The prevalence of *current* smoking and *ever* smoking over time in one 5-years and seven 10-years generations was firstly examined graphically for participants who participated in at least two waves. The smoking prevalence within each generation as measured every wave was plotted against the mean age of the participants. A line linking the smoking prevalences for each generation represents the change in current or ever smoking prevalence with ageing. A generation shift is defined as the difference in the age-related smoking prevalence of two generations (two lines).

Logistic regression was used to statistically test generation shifts by modelling current smoking (or ever smoking) as a function of age, generation and their interaction. In this way a different age effect for each generation can be modelled. Furthermore, random effects were added to model the serial correlation of the repeated measurements within the individuals. All analyses were stratified by sex. Generation shifts in the prevalence of current and ever smoking between two generations having reached the same age were tested at predefined mean ages: 35, 45, 55, 65 and 75 years for successive generations (LASA & DCS) and 45 and 55 for non-successive generations (DCS only). Generation shifts could not be modelled for the oldest generation (predefined mean age 85) since no smokers were left at the last three follow-up measurements. Statistical significance (p < 0.05) of generation shifts was tested by the significance of the interaction terms between age and generation. Modelled prevalence rates of smoking at predefined mean ages were derived from the model with the estimate statement. Interaction terms between education and generation were used to test whether differences between generations differed significantly between educational levels. All analyses were performed in SAS version 9.3 (SAS Institute, Cary, North Carolina, USA).

## Results

At baseline, there were more women than men within each 10-year generation and mean ages were comparable between the two sexes (Table 1, not statistically tested). The number of individuals with a low level of education was higher among older generations.

**Table 1:** **Baseline characteristics of the Doetinchem Cohort Study (DCS) and the Longitudinal Aging Study Amsterdam (LASA) for men and women separately**

**Age of generation at baseline of the DCS**	**20-29 y**		**30-39 y**		**40-49 y**		**50-59 y**	
	**Men**	**Women**	**Men**	**Women**	**Men**	**Women**	**Men**	**Women**
N (%)	467 (43)	618 (57)	941 (46)	1084 (54)	926 (49)	957 (51)	658 (47)	732 (53)
Age	24.9	24.9	34.6	34.4	43.8	43.7	54.0	54.1
	(3.0)	(2.8)	(2.9)	(2.8)	(2.6)	(2.8)	(2.8)	(2.9)
Level of education, N (%)								
• Low	244 (52)	302 (49)	463 (49)	694 (64)	542 (59)	724 (76)	403 (61)	601 (83)
• Middle	173 (37)	251 (41)	257 (28)	207 (19)	189 (20)	110 (12)	112 (17)	66 (9)
• High	52 (11)	65 (10)	217 (23)	180 (17)	190 (21)	119 (12)	142 (22)	61 (8)
Smoking, N (%)								
• Current	186 (40)	237 (38)	347 (37)	409 (38)	310 (34)	318 (33)	204 (31)	182 (25)
• Ex	59 (12)	108 (18)	283 (30)	335 (31)	361 (39)	244 (26)	307 (47)	137 (19)
• Never	224 (48)	273 (44)	309 (33)	339 (31)	253 (27)	395 (41)	147 (22)	413 (56)
Packyears	3.3	3.0	7.8	6.6	13.4	7.9	17.9	7.6
	(5.2)	(3.9)	(8.5)	(7.3)	(13.5)	(10.1)	(16.7)	(12.4)
**Age of generation at baseline of the LASA**	**55-59 y**		**60-69 y**		**70-79 y**		**80-85 y**	
	**Men**	**Women**	**Men**	**Women**	**Men**	**Women**	**Men**	**Women**
N (%)	153 (48)	168 (52)	355 (47)	400 (53)	305 (48)	326 (52)	118 (46)	136 (54)
Age	57.6	57.7	64.9	65.0	75.1	75.0	82.5	82.7
	(1.4)	(1.4)	(2.9)	(2.8)	(2.9)	(2.9)	(1.5)	(1.5)
Level of education, N (%)								
• Low	68 (45)	98 (58)	161 (46)	270 (67)	173 (57)	224 (69)	76 (64)	99 (73)
• Middle	51 (33)	52 (31)	125 (35)	102 (26)	85 (28)	81 (25)	21 (18)	23 (17)
• High	34 (22)	18 (11)	68 (19)	28 (7)	47 (15)	21 (6)	21 (18)	14 (10)
Smoking, N (%)								
• Current	64 (42)	45 (27)	110 (31)	75 (19)	96 (31)	36 (11)	28 (24)	12 (9)
• Ex	77 (51)	61 (36)	218 (61)	135 (34)	180 (59)	90 (28)	86 (73)	28 (21)
• Never	11 (7)	62 (37)	27 (8)	190 (47)	29 (10)	200 (61)	4 (3)	96 (70)
Packyears	27.4	11.7	27.4	8.0	25.2	4.9	25.0	2.5
	(26.0)	(19.1)	(27.0)	(15.2)	(25.5)	(13.1)	(24.8)	(11.6)

Mean and standard deviation are presented for continuous variables, otherwise indicated.

### Current smoking

**Figure 1:**
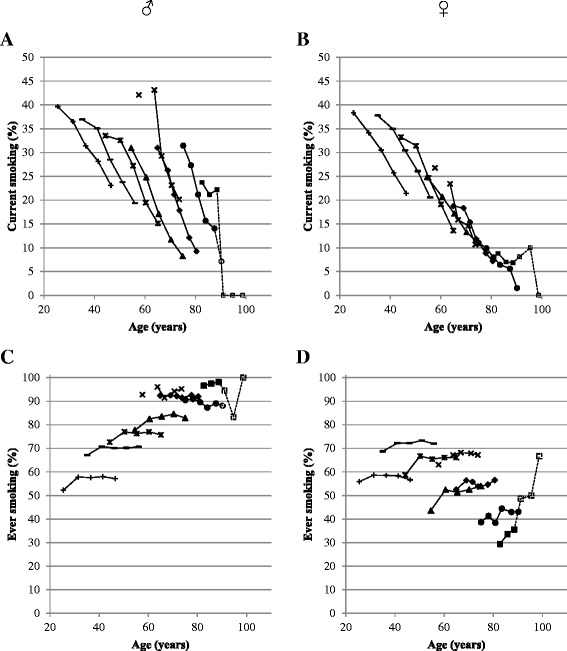
**Change in prevalence of smoking (A and B) and ever smoking (C and D) with ageing during follow-up in generations aged 20–29 (− + −), 30–39 (−─–), 40–49 (−**✻–), 50–59 (−▲–), 55–59 (− × −)*, 60–69 (−♦–), 70–79 (−●–) and 80–89 (−■–) years at baseline for men (A and C) and women (B and D) separately. Open markers and dotted lines represent groups consisting of less than 50 participants. *During the second wave of the LASA study, participants aged 55–59 at baseline were not invited for the medical interview, which explains the missing link between the first and the third data point for this generation.

**Figure 2:**
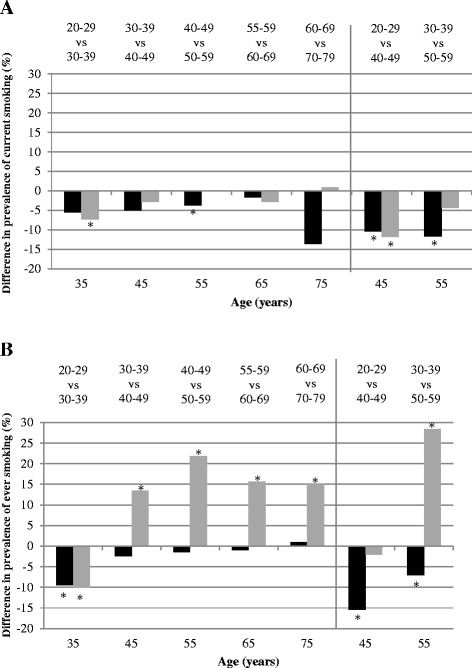
**Raw generation shifts in the prevalence of current smoking (A) and ever smoking (B) between successive (left panel) and non-successive (right panel) generations of men (■) and women (**  **).** An asterisk (*) indicates a significant (P < 0.05) generation shift. A negative difference in current or ever smoking between two generations indicates that the more recently born generation, at the same age, smoked less often than the older one.

With ageing, the prevalence of current smoking decreased among all generations (Figure 1A and B). Favourable generation shifts, i.e. every more recently born generation smoking less, were present among men, except for those aged 55–59 at baseline as compared to those aged 60–69 at baseline. In the most recently born male generation of the DCS, aged 20–29 years at baseline (1987–1991), a prevalence of current smoking of 31.4% was observed as they reached a mean age of 35 in 1998–2002 (Figure 1A, third data point). The prevalence of current smoking of the generation aged 30–39 at baseline (also at mean age 35) was higher: 37%, which shows a generation shift of −5.6% over a period of 10 years. This difference was however not statistically significant, which can be seen in Figure 2 where all shifts between two generations are plotted. Even though most more recently born generations of men smoked less often than their predecessors (Figure 1A), only a third of the comparisons between successive generations of men (Figure 2A) were statistically significant, but among non-successive generations, both comparisons were significant. The prevalence of current smoking was 10.5% lower (33.6% vs. 23.1%) in men originally aged 20–29 years old at baseline, but now aged 45 on average, compared to men aged 40–49 years old at baseline. At age 55, current smoking prevalence was 11.7% lower (31% vs. 19.3%) comparing those aged 30–39 years at baseline with those aged 50–59 years baseline.

In women, a generation shift in current smoking was observed between the two most recently born generations at age 35, since the two lines did not cross each other (Figure 1B), and this shift (−7.3%) was also statistically significant (Figure 2A). Between non-successive generations, a generation shift of −11.8% was observed between 20–29 years olds at baseline and 40–49 years olds at baseline, when both generations were aged 45 (Figure 2A).

### Ever smoking

Among men, a significant generation shift between successive generations was only observed between 30–39 years olds at baseline and those aged 30–39 ten years later (Figure 1C), a reduction of 9.5% (67.1% vs. 57.6%). Between non-successive generations, favourable generation shifts were more pronounced, with a reduction of 15.3% between those aged 20–29 at baseline with those aged 40–49 at baseline, at age 45 (Figure 2B).

Large unfavourable generation shifts in ever smoking were found among women (Figure 1D): the prevalence of ever smoking increased with at least 10% in every successive more recently born generation. One exception is the youngest generation (20–29 years at baseline), which showed after ten years a lower prevalence of ever smoking (10.1% lower) as compared to the 30–39 years olds at baseline. The increase in ever smoking was most pronounced between non-successive generations of women, where an increase of 28.5% at age 55 was found between women aged 50–59 years at baseline and women with the same age twenty years later (Figure 2B).

### Smoking by education

Generation shifts in the prevalence of current and ever smoking stratified by educational level are shown in Table 2. The generation shift in *ever* smoking among the youngest successive generations of men (−9.5%) was found in the high (−24.3%) and middle (−10.6%) educated, but not in the low educated (+4.5%). In non-successive generations, the overall favourable generation shifts in the prevalence of *current* and *ever* smoking were found in middle and high educated men at age 45, but not in the low educated. For the youngest generations, generation shifts in *current* and *ever* smoking were significantly different in low educated compared to middle and high educated (footnotes b&c Table 2).

**Table 2:** **Sex and education-stratified observed and modelled** ^**ǂ**^ **(between parenthesis) differences in current smoking and ever smoking between successive and non-successive generations**

**Differences between generations**	**20-29**	**30-39**	**40-49**	**55-59**	**60-69**	**20-29**	**30-39**
	**vs**	**vs**	**vs**	**vs**	**vs**	**vs**	**vs**
	**30-39** ^**1**^	**40-49** ^**1**^	**50-59** ^**1**^	**60-69** ^**2**^	**70-79** ^**2**^	**40-49** ^**3**^	**50-59** ^**3**^
Difference (%) at age	35	45	55	65	75	45	55
Men overall							
Current smoking	−5.6 (−3.8)^4^	−5.0 (−3.0)	−3.8 (−4.3)*	−1.7 (+1.4)	−13.6 (−3.2)	**−10.5 (−6.0)** ^**4**^ *	−11.6 (−5.6)*
Ever smoking	−9.5 (−9.8)**	−2.5 (−2.6)	−1.5 (−1.4)	−1.0 (+0.1)	+1.0 (+2.3)	−15.3 (−11.0)**	−7.0 (−4.6)*
Men by education							
*Low*							
Current smoking	−0.3 (+1.5)	+0.6 (+3.2)	−11.2 (−10.3)**	−1.6 (+2.1)	−18.7 (−4.8)	+0.1 (+5.0)	−13.1 (−6.0)
Ever smoking	+4.5 (+1.7)	−3.5 (−2.4)	−0.4 (−0.9)	−8.8 (−6.9)	+3.2 (+3.6)	−2.4 (−0.1)	−7.0 (−3.7)
*Middle*							
Current smoking	−5.1 (−4.1)	−5.3 (−4.0)	+1.7 (+0.6)^b^	+7.4 (+6.7)^a^	−1.6 (+2.0)	−9.2 (−7.4)* ^**b**^	−7.1 (−1.7)
Ever smoking	−10.6 (−13.8)** ^**b**^	+0.5 (−1.9)	−5.6 (−4.8)	+8.2 (+8.4)^ab^	+0.4 (+1.8)	−16.1 (−13.9)** ^**b**^	−6.3 (−7.3)
*High*							
Current smoking	−10.3 (−9.6)* ^**c**^	−7.2 (−6.8)* ^**c**^	+8.6 (+4.9)^c^	−14.2 (−7.6)^a^	−18.6 (−6.5)*^a^	−21.4 (−14.6)** ^**c**^	−2.9 (+0.4)
Ever smoking	−24.3 (−21.3)** ^**c**^	−0.4 (−1.1)	+3.4 (+3.5)	+1.2 (+1.9)^a^	+0.5 (+2.6)^a^	−26.3 (−21.2)** ^**c**^	−3.9 (+1.5)
Women overall							
Current smoking	−7.3 (−6.2)*	−2.8 (−1.9)	−0.2 (−0.1)	−2.9 (+0.6)	+0.9 (+4.4)	−11.8 (−6.6)*	−4.3 (+0.3)
Ever smoking	−10.1 (−10.9)**	+13.4 (+12.4)**	+21.9 (+20.6)**	+15.7 (+12.6)*	+15.1 (+16.7)**	−2.1 (+0.7)	+28.5 (+31.1)**
Women by education							
*Low*							
Current smoking	−2.2 (−0.4)	−3.7 (−1.4)	+1.6 (+2.4)	+1.6 (+5.4)	−0.9 (+2.9)	−6.7 (−0.8)	−2.2 (+3.4)
Ever smoking	−1.3 (−3.5)	+16.8 (+15.9)**	+20.7 (+22.7)**	+20.0 (+17.5)*	+12.3 (+16.3)**	+7.3 (+11.1)*	+36.5 (+36.1)**
*Middle*							
Current smoking	−11.3 (−11.2)** ^**b**^	+3.5 (+1.2)	−5.3 (−7.7)^b^	−15.6 (−11.2)* ^**ab**^	+3.7 (+6.5)	−10.9 (−7.9)*	−8.4 (−4.7)
Ever smoking	−17.1 (−18.1)**	+11.9 (+9.9)*	+22.6 (+16.6)*	+7.4 (+2.7)^a^	+17.3 (+15.0)*	−8.5 (−9.1)* ^**b**^	+24.5 (+24.3)**
*High*							
Current smoking	−5.6 (−4.4)	−2.0 (−2.0)	+3.7 (+2.4)	+2.1 (+0.4)^a^	+6.5 (+9.3)^a^	−8.7 (−5.1)	+0.7 (+2.3)
Ever smoking	−12.2 (−11.8)*	+7.7 (+7.6)	+20.3 (+10.8)	−0.8 (−5.2)^a^	+23.6 (+22.9)^a^	−9.3 (−5.9)^c^	+17.5 (+16.5)* ^**c**^

^1^The oldest generation reached a mean age of 35, 45 or 55 years in 1987–1991 and the youngest generation in 1998–2002.

^2^The oldest generation reached a mean age of 65 or 75 years in 1992–1993 and the youngest generation in 2001–2002.

^3^The oldest generation reached a mean age of 45 or 55 years in 1987–1991 and the youngest generation in 2008–2012.

^4^A negative difference in current or ever smoking between two generations indicates that the younger generation smoked less often than the older one.

*p < 0.05, **p < 0.001.

^a^Comparison based on groups with less than 50 observations.

^b^Difference in smoking prevalence between generations differed significantly between the middle educated compared to the low educated (tested by interaction education*generation).

^c^Difference in smoking prevalence between generations differed significantly between high educated compared to low educated (tested by interaction education*generation).

^ǂ^Model: For current smoking and ever smoking a logistic random effect model was fitted with generation, education, age, age*generation and education*generation.

Among women, the (overall) generation shifts in *current* smoking among the youngest successive generations was only observed among middle educated women (reduction 11.3%; 39.1% vs. 27.8%). Unfavourable generation shifts in *ever* smoking in women were mainly observed at ages 45, 55, 65, 75 and 85 in more recently born generations of low educated women (+16.8%, +20.7%, +20.0%, +12.3% and +10.1%), less often in middle educated women (at age 45, 55 and 75), and not significantly in high educated women. These unfavourable shifts among the low educated were also shown between non-successive generations (+7.3% at age 45 and +28.5 at age 55), whereas among the middle and high educated the prevalence of *ever* smoking increased less or even decreased. The statistically significant overall decrease in ever smoking between the youngest successive generations of women (−10.1% at age 35) was observed among high (−12.2%) and middle educated women at age 35 (−17.1%), but not in low educated women. These generation shifts in *ever* smoking were significantly different in low educated compared to both middle and high educated, but most differences between the educational levels in women were non-significant.

## Discussion

In both men and women, the most recently born generations showed the most favourable smoking figures. For men, this is a continuation of the decreasing trend of smoking, especially in middle and high educated men. For women this means there is a break in the unhealthy trend of each younger generation showing more unfavourable figures for ever smoking. Therefore, the burden of smoking-related diseases is expected to decrease when currently young generations of men grow old, although for women, this decrease seems only to have started in the youngest generation. The future burden of smoking-related diseases is still expected to increase because - with the exception of the youngest generations - all more recently born generations of women smoked more often than their predecessors.

The generation shifts in current smoking found in the literature show favourable figures for both men and women in Great-Britain [5]. In Sweden [3] and Finland [12] the smoking prevalence was lower in more recently born men compared to their predecessors, but not in more recently born generations of older women, which is similar to our results. We found favourable generation shifts among men in ever smoking, especially among the higher educated groups when comparing non-successive generations. The most obvious unfavourable generation shifts were shown in the low educated women. This is in line with what is found in Italy [7] and Finland [12], where ever smoking rates in more recently born generations of low educated women were higher compared to more recently born generations of high educated women. Educational inequalities in smoking are well-known in European countries [1,2,13] and this study confirms that the lower educational groups continue to do worse.

The observed generation shifts in smoking can be explained with the sex- and socioeconomic-specific theory of diffusion of smoking in developed countries [16,17]. This theory describes the diffusion of smoking throughout society in the 19^th^-21^th^ century in four stages. Smoking was first taken up by men of higher socioeconomic status (stage 1), followed by a diffusion of smoking towards men of lower and women of higher socioeconomic status (stage 2). Stage 3 marked the decrease of smoking among men - due to emerging knowledge on smoking-related diseases - while women’s smoking prevalence was peaking. During the fourth stage, smoking rates declined for men and women though specifically for higher socio-economic groups, so that educational differences in smoking are now widening. The results of the present study suggest that the Netherlands was in the 3^rd^ stage at the start of the data collection and in transition to the 4^th^ stage of the smoking epidemic during the study period.

The generation shifts shown were mainly due to differences in (changes in) smoking status – starting or quitting smoking - between generations, but can also be due to selective mortality, especially among the higher age groups. Smokers have an increased risk of mortality [18] but mortality rates of smokers and non-smokers usually start to diverge around the age of 70 [19]. Therefore, generation shifts as we observed between the more recently born generations of men and women are not yet likely to be affected by selective mortality. For older generations at baseline, this selective mortality might play a role, but for those generations there is also the largest impact of selective participation and selective loss to follow-up. In general, participants of health surveys are healthier and higher educated than non-participants [20]. Our prevalences of smoking may be a slight underestimation of the actual smoking prevalence, but we expect that the (direction of) change over time in smoking prevalence is less affected. However, study attrition due to physical and cognitive health problems is of course higher in the older generation, so the presented smoking figures in the older generations (as indicated by the dotted lines in Figure 1) should be interpreted carefully. In general, some age-specific selection among study participants will exist but at this moment, we cannot quantify this selection bias.

Besides selection bias, another limitation refers to a potential time trend in the quality of the response to questions on smoking, because there has been a change in what is socially acceptable. We did not find literature that can aid us to quantify this. However, in our study only a very small proportion of the participants (2.5%) responded as ‘never smoking’ while earlier reported ‘former smoking’ or ‘current smoking’. This was corrected prior to the analyses by changing ‘never smoking’ to ‘former smoking’ for these participants.

The strength of the current study is that we used two population-based, prospective cohort studies with high response rates (75-82%), a long follow-up and broad age range. Generations were defined at baseline as groups of individuals of the same age and the same persons were followed up for many years. This reduced between-subject variations compared to studies using multiple cross-sectional surveys to study generation shifts (pseudo- or constructed birth cohort studies).

## Conclusion

Generation shifts in smoking will result in generation shifts in smoking-related diseases: with all else remaining the same, the old age incidence of lung cancer and COPD is expected to be much lower in generations which are now in their twenties or thirties. Also the occurrence of cardiovascular diseases (especially coronary heart diseases), nasopharyngeal tumours and other smoking related negative health effects [21,22] may be affected by these changing smoking rates. Besides disease occurrence, decreased mortality from cardiovascular diseases has been shown to be primarily attributed to decreased smoking rates [23]. It takes, however, a substantial time for these changes to become apparent in the health status of the population, because the most prominent chronic changes will begin to emerge at ages > 70 years [23]. The expected changes in health status are also specific for sex and socioeconomic status; men doing better than women, and lower educated doing worse than higher educated. For women, our results predict that there will be an increase in smoking-related health problems in the coming decades, but a decrease in the long run seems to be announced by the lower smoking figures among the most recently born generations. Those belonging to the lower educated groups in the Netherlands are still a major reason for concern. Due to the overall reduced smoking prevalence, issues of tobacco control may not have been a priority from the view of policy makers [24]. However, the fact that cigarette smoking has become more concentrated among the lower educated ensures that anti-smoking policies remain relevant and should especially target persons with a low educational level. These policies should focus on preventing people from starting to smoke, preferably at young age, since 88% of smokers start smoking before the age of 18 [21].

## Abbreviations

DCS: Doetinchem Cohort Study
LASA: Longitudinal Aging Study Amsterdam
COPD: Chronic Obstructive Pulmonary Disease
